# Microvascular Capillary and Precapillary Cardiovascular Disturbances Strongly Interact to Severely Affect Tissue Perfusion and Mitochondrial Function in Myalgic Encephalomyelitis/Chronic Fatigue Syndrome Evolving from the Post COVID-19 Syndrome

**DOI:** 10.3390/medicina60020194

**Published:** 2024-01-23

**Authors:** Klaus Josef Wirth, Matthias Löhn

**Affiliations:** Institute of General Pharmacology and Toxicology, University Hospital Frankfurt am Main, Goethe-University, Theodor-Stern Kai 7, D-60590 Frankfurt am Main, Germany; wirth@em.uni-frankfurt.de

**Keywords:** myalgic encephalomyelitis/chronic fatigue syndrome, post-COVID-19 syndrome, long COVID, precapillary cardiovascular disturbances, capillary disturbances, ß2AdR dysfunction, mitochondrial dysfunction, post-exertional malaise, exercise intolerance

## Abstract

Myalgic Encephalomyelitis/Chronic Fatigue Syndrome (ME/CFS) is a frequent, debilitating and still enigmatic disease. There is a broad overlap in the symptomatology of ME/CFS and the Post-COVID-19 Syndrome (PCS). A fraction of the PCS patients develop the full clinical picture of ME/CFS. New observations in microvessels and blood from patients suffering from PCS have appeared and include microclots and malformed pathological blood cells. Capillary blood flow is impaired not only by pathological blood components but also by prothrombotic changes in the vascular wall, endothelial dysfunction, and the expression of adhesion molecules in the capillaries. These disturbances can finally cause a low capillary flow and even capillary stasis. A low cardiac stroke volume due to hypovolemia and the inability of the capacitance vessels to adequately constrict to deliver the necessary cardiac preload generate an unfavorable low precapillary perfusion pressure. Furthermore, a predominance of vasoconstrictor over vasodilator influences exists, in which sympathetic hyperactivity and endothelial dysfunction play a strong role, causing the constriction of resistance vessels and of precapillary sphincters, which leads to a fall in capillary pressure behind the sphincters. The interaction of these two precapillary cardiovascular mechanisms causing a low capillary perfusion pressure is hemodynamically highly unfavorable in the presence of a primary capillary stasis, which is already caused by the pathological blood components and their interaction with the capillary wall, to severely impair organ perfusion. The detrimental coincidence of microcirculatory and precapillary cardiovascular disturbances may constitute the key disturbance of the Post-COVID-19 syndrome and finally lead to ME/CFS in predisposed patients because the interaction causes a particular kind of perfusion disturbance—capillary ischemia/reperfusion—which has a high potential of causing mitochondrial dysfunction by inducing sodium- and calcium-overload in skeletal muscles. The latter, in turn, worsens the vascular situation through the generation of reactive oxygen species to close a vicious cycle from which the patient can hardly escape.

## 1. Introduction

Myalgic Encephalomyelitis/Chronic Fatigue Syndrome (ME/CFS) is a frequent, debilitating disease and is associated with a number of syndromes or conditions, including postural orthostatic tachycardia syndrome (POTS) [1,2], orthostatic intolerance (OI) [3], and small fiber neuropathy (SFN) [1]. ME/CFS shows a broad overlap of symptomatology with the Post-COVID-19 Syndrome (PCS). A fraction of the PCS patients develop the full picture of ME/CFS to strongly raise the prevalence of ME/CFS (designated as PCS-ME/CFS) [4].

Investigations on the causes of long COVID and PCS have identified pathological blood components—microclots and malformed blood cells—in patients after COVID-19 infection. Lymphocytes had a significantly decreased stiffness, monocytes and neutrophils were increased in cell size, erythrocytes were less deformable, and reduced natural killer cell function was also found [5,6,7,8,9,10]. These pathological blood cells and microclots can impede capillary blood flow and even induce capillary stasis, and thus significantly worsen organ blood supply. Capillary blood flow is not only impaired by pathological blood components but also by changes in the vascular wall, such as endothelial dysfunction (ED) and the expression of adhesion molecules in the capillaries, which leads to an enhanced adhesivity of blood components to the vessel wall to aggravate the flow disturbance.

Various cardiovascular disturbances and abnormalities have been known for quite a long time in ME/CFS before COVID-19, including hypovolemia, low stroke volume, and high vasoconstrictor tone [1,10,11,12,13]. Similar cardiovascular disturbances have been found in PCS or PCS-ME/CFS [1,3,10,11,12,14,15,16]. To delineate them from the newly discovered findings of pathological blood components that must disturb capillary flow, we use the term precapillary cardiovascular disturbances. Separately or jointly, these different precapillary cardiovascular disturbances have the same effect. They lower the capillary perfusion pressure. In a direct comparison, long COVID and ME/CFS patients were found to exhibit similarly impaired endothelial function, indicating a potential vascular involvement in the pathogenesis of these post-viral illnesses [17].

In the presence of a reduced capillary flow velocity caused by the pathological blood components, the inflammatory capillary wall changes, and the interaction between the two, a physiological or a high capillary pressure, are of utmost importance to prevent capillary stasis. The single hemodynamic disturbances and their detrimental interactions, which severely affect microcirculation and organ blood flow, will be highlighted in detail in this paper. We raise the hypothesis that the coincidence or interaction of both disturbances—primary microcirculatory capillary disturbances and precapillary cardiovascular disturbances—constitutes the key disturbance of the Post-COVID-19 syndrome and finally leads to ME/CFS in predisposed patients.

Microvascular capillary disturbances in ME/CFS of causes other than PCS have not been published apart from a report of diminished red blood cell deformability in ME/CFS [7], but investigations are ongoing. Therefore, we limit our conclusions here to ME/CFS developed out of PCS (PCS-ME/CFS).

## 2. Precapillary Cardiovascular Disturbances and Capillary Disturbances in ME/CFS and PCS—The Causes and Effects of Hypovolemia and Low Stroke Volume

**Figure 1:**
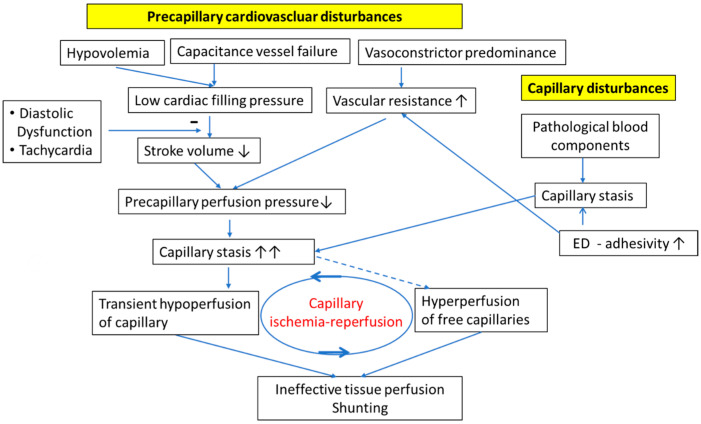
Association of precapillary cardiovascular disturbances and capillary disturbances in ME/CFS and PCS. Capillary low flow or even stasis is triggered by malformed blood components, such as microclots, malformed leukocytes, and an increased endothelial adhesivity promoted by increased expression of adhesion molecules in the capillaries. Capillary stasis is strongly aggravated by the precapillary cardiovascular disturbance, generating a low capillary perfusion pressure for which a low stroke volume and high vasoconstrictor tone account for. The low stroke volume is caused by both hypovolemia and failure of capacitance vessels to adequately constrict to raise the circulating blood volume. A peculiar perfusion disturbance—capillary ischemia/reperfusion—occurs and has a high pathogenetic potential. ED: endothelial dysfunction. arrows down and minus mean inhibition; arrows up mean promotion.

The term “precapillary cardiovascular disturbances” includes different cardiovascular disturbances, hypovolemia, capacitance vessel failure, and peripheral vasoconstrictor predominance (Figure 1). Hypovolemia and capacitance vessel failure either independently or jointly reduce cardiac filling pressure. As a result of low ventricular filling, stroke volume and cardiac output significantly decrease leading to a decrease in tissue perfusion and capillary perfusion pressure. Surprisingly, cardiac output was found increased in a group of ME/CFS patients [18], but this was most likely due to left-to-right shunting diverting blood flow from skeletal muscles to other organs (steal effect). The causes of shunting are discussed in Section 3, Section 5, and Section 6. The causes for hypovolemia are thought to be renal hyperexcretion with low renin (renin paradox) and microvascular leakage. These disturbances have been dealt with in detail in a previous paper [16]. We hypothesize that the poor energetic situation in skeletal muscle as the result of poor perfusion and mitochondrial dysfunction leads to the excessive, compensatory production of vasoactive mediators that cause renal hyperexcretion and microvascular leakage to explain hypovolemia [16], as described in Section 6.

Concerning dysfunction of the capacitance vessels, their contractile dysfunction could be due to three different mechanisms from a theoretical point of view:

(1) Autonomic dysfunction or damage to sympathetic nerves could be a potential cause of ineffective contraction.
(2) Primary dysfunction due to structural or functional disturbances of the capacitance vessels. Connective tissue disorders, also affecting the vasculature, such as the Ehlers–Danlos Syndrome (EDS) and Marfan Syndrome are related to ME/CFS [19,20,21]. In the latter, large vessels are distended. In EDS, peripheral veins seem visibly distended [22,23]. Orthostatic intolerance appears early on in patients with PCS [24,25,26]. In a previous publication, we hypothesized that SARS-CoV-2-infection affects capacitance vessels, mainly veins, directly by the virus itself or via cytokines and/or indirectly via disturbed microcirculation of the vasa vasorum of these vessels (microcirculatory disturbance) to cause orthostatic intolerance early on after the acute infection [3].
(3) There may be a failure in appropriate regulation at the vascular level. The latter could be disturbed by an impairment of vasoconstrictor mechanisms (A) or by an excess of vasodilator mechanisms (B).
(3A) Autoantibodies against different vasoregulators have been found, including autoantibodies against alpha1-adrenergic receptors, which is the main mechanism of venous contraction [27,28].
(3B) Histamine: the high prevalence of allergies and mast cell overactivation, which might even be augmented by SARS-CoV-2-infection, particularly in the gut, and the fact that histamine has a strong vasodilator effect on veins in humans [3,15,29,30,31] warrant further discussion. The most convincing evidence for the involvement of histamine is the alleviating effect of long-term symptoms of Post-COVID-19 infection by anti-histamine treatment [32,33,34,35,36]. From a theoretical point of view, the dilating effect of histamine on human veins may be particularly detrimental if the capacitance vessels or veins are already weakened or structurally distended as in EDS or Marfan Syndrome, or damaged by the mechanisms after COVID-19 infection [15]. For physical (Laplace’s law) and physiological reasons (lacking overlap of sarcomeres), the force needed for effective constriction is higher in overstretched circular structures. Histamine induces microvascular leakage. The resulting loss of plasma volume may contribute to a low cardiac filling pressure. The physiological mechanisms of venous constriction by alpha1-adrenergic stimulation required for appropriate orthostatic function and for raising the circulatory blood volume for exercise may then become quite ineffective against two simultaneously present disturbances that weaken venous contraction. Additionally, autoantibodies against alpha1-adrenergic receptors may even weaken the constrictor stimulus.

Taken together, key mechanisms which generate a low precapillary perfusion pressure are hypovolemia and the contractile disturbance of capacitance vessels. Separately or jointly, they lead to a low cardiac preload and subsequently low stroke volume. In the presence of a low cardiac filling pressure, both tachycardia, by shortening the cardiac filling time, and diastolic dysfunction worsen ventricular filling, which will be further discussed below.

### The Disturbed Vasoconstrictor/Vasodilator Balance Causes Excessive Vasoconstriction

In ME/CFS, there is vast literature showing vasoconstrictor predominance over vasodilator influence due to a high sympathetic and low vagal tone, as well as endothelial dysfunction (ED) occurring via a different mechanism that raises vascular resistance to which resistance vessels make the strongest contribution (Figure 1) [37]. This leads to a pressure fall behind the resistance vessels with the consequence that capillary perfusion and capillary perfusion pressure become low.

Regarding the interaction of disturbed cardiac filling with increased vascular resistance, it cannot be emphasized enough that the combined effect of low stroke volume together with a high vascular resistance has a detrimental effect by strongly lowering tissue perfusion and capillary perfusion pressure.

## 3. Microcirculatory Disturbances in ME/CFS and PCS

Low capillary perfusion pressures, as outlined above as the result of the precapillary vascular disturbances, coincide with preexisting capillary stasis. The interaction of these disturbances has further detrimental effects on capillary blood flow (Figure 1). Microcirculatory disturbances and capillary stasis are primarily caused by the presence of pathological blood components mainly too large so that they hinder blood flow and induce prothrombotic changes in the capillary wall (increased adhesivity). There is also a detrimental interaction of pathological blood components with prothrombotic, inflammatory capillary wall changes. The stagnation or slow flow of blood cells enhances the interaction and time for interaction with the prothrombotic wall changes, and the mainly expressed adhesive molecules, further slowing blood flow velocity [9]. The observations of pathological blood components include microclots, large cells, less deformable erythrocytes, decreased lymphocyte stiffness, increased monocyte cell size, large, deformable, activated neutrophils, activated monocytes and platelets. These activated cells may convert the normally antithrombotic surface of the vascular endothelium to a prothrombotic state to upregulate coagulation and stimulate inflammatory reactions (thrombo-inflammation) [5,9,38]. Independent of the effect of inflammatory blood cells on the microvessels, the vascular system, including capillaries, could be directly damaged by the SARS-CoV-2 virus or affected by cytokines, generating an inflammatory and prothrombotic vascular wall [9,10].

Due to the long-term inflammatory environment in long COVID (PCS), immune cells, such as neutrophils, are excessively activated and persistently degranulated, maintaining inflammatory responses. This leads to the formation of fibrin amyloid microclots, which promote tissue hypoxia and impaired oxygen exchange and may additionally block the capillaries [9,10,39,40,41]. It has been described that chronic inflammation can trigger coagulation proteases to bind to protease-activated receptors on the activated endothelium to induce the synthesis and expression of cell adhesion molecules, which further promotes microcirculatory disorders [9]. Several endothelial cell-related biomarkers have been observed to be strongly correlated with COVID-19 and PCS, such as VWF and Factor VIII, as well as ET-1 and angiopoietin-2 [9]. A recent study identified a disturbed retinal microcirculation to be strongly associated with PCS and ME/CFS, which may serve as a potential marker for microcirculatory disorders [10,42]. Notably, the thrombo-inflammatory status persists months after the patient has recovered and the virus has cleared [5,40,43]. Therefore, malformed blood cells, microclots, and thrombo-inflammation can potentially impede capillary perfusion and promote a microcirculatory disorder and thus impair organ blood supply via capillary obstruction [9].

It is unlikely that microclots, with a size larger than their capillary diameter, play a major role in capillary perfusion disorder since these microclots would get stuck in an organ and thus be removed from circulating blood with every circulatory passage. If very large microclots were common, one would expect the development of ischemic organ damage by vascular occlusions over time, or the development of pulmonary hypertension if they got stuck in the pulmonary vascular system [5,40].

Although severe ischemic organ damage has not been observed in PCS-induced ME/CFS so far, the permanent obstruction of capillaries, e.g., by microclots larger than their capillary diameter, may still occur and could account for the observation of vessel rarefaction (disappearance of capillaries) in skeletal muscle [44,45]. In a recent muscle biopsy study, patients with post-COVID exertion intolerance had fewer capillaries, thicker capillary basement membranes, and increased numbers of CD169^+^ macrophages [44].

It is likely that transient capillary blood stasis is present in a fraction of the capillaries so that the free capillaries are hyperperfused, which would lead to a blood flow distribution disorder and capillary shunting (Figure 1). Peripheral left-to-right shunting was indeed observed in patients with ME/CFS [18]. After removal of the obstruction, the transiently ischemic capillary would be reperfused, generating the phenomenon of capillary ischemia/reperfusion. Post-ischemic hyperperfusion may contribute to capillary shunting. Other probably more important causes for the observation of shunting during exercise in some patients are discussed in Section 5 and Section 6.

Ischemia/reperfusion is a pathomechanism with a high potential of causing a particular type of damage that will be extensively explained in Section 5. Given the variety in the size of microclots and of disturbed blood cells, all degrees of obstruction ranging from weak slowing of capillary flow velocity to transient ischemia seem possible and could occur simultaneously in different capillaries. Microvascular rarefaction or temporary obstruction markedly alter blood flow distribution, more significantly than microvascular constriction [46,47]. These various disturbances might also have a significant impact on neurovascular coupling, a mechanism which is based on vasodilator release by active nerve cells to increase their energy supply by dilating local capillaries and precapillary sphincters, which protect capillaries from high blood pressure while maintaining blood flow [48,49,50]. Cerebral blood flow measured over a large cerebral artery was found decreased in ME/CFS and PCS [51]. The extent of flow reduction measured over a large brain artery does not reflect the true extent of the disturbance as it does not consider the microcirculatory disturbance and the maldistribution of blood flow in the capillary system (capillaries with no flow versus hyperperfused capillaries). The severity of the resulting cerebral blood flow disturbance may explain cognitive impairment, mental fatigue, and, at least partially, brain fog. Endothelial dysfunction found in ME/CFS and PCS certainly worsens the function of capillaries as well as of precapillary vessels, e.g., by raising the vasoconstrictor tone of resistance vessels [17,52,53].

## 4. The Interaction of the Precapillary Cardiovascular Disturbances with the Capillary Disturbances—A Highly Unfavorable Synergistic Interaction in ME/CFS and PCS

To quickly overcome a capillary stasis, the precapillary perfusion pressure must be high, but it is lower than normal in PCS and ME/CFS. Because it is very likely that only a fraction of the capillaries is transiently occluded and ischemic, e.g., via microclot-induced blood stasis, other capillaries are hyperperfused and a blood flow distribution disorder may develop (capillary shunting) (Figure 1). In this situation, the low stroke volume cannot maintain a continuous capillary flow, particularly during diastole. Physiologically, capillary blood flow is continuous, also in diastole, since the energy delivered in systole is stored in the vascular system (referred to as “Windkessel-effect”). Interestingly, a recently performed clinical study used carotid–femoral pulse wave velocity as a measurement to predict COVID-19-related cardiovascular complications and found a marked increase in arterial stiffness and an increased carotid–femoral pulse wave velocity [54]. An increase in arterial stiffness is due to a structural loss of vascular elasticity or due to a high tone of conductance vessels to diminish the Windkessel function. The latter is not only caused by the elastic storage of kinetic energy provided by the systole but also by a dynamic phenomenon—by the reflection of pulse waves that are appropriately timed to the diastole. An increase in arterial stiffness, therefore, is not only indicative of a loss of elasticity but also changes the time pattern of pulse wave reflection so that reflection occurs earlier, leading to a loss of capillary thrust at least later in diastole [55,56]. Therefore, the determination of the carotid–femoral pulse wave velocity could potentially serve as a biomarker for COVID-19-related cardiovascular complications. Another clinical study on 256 patients suffering from ME/CFS showed a decreased cognitive efficiency, a reduced narrow pulse pressure less than 25% of systolic pressure, and an increased heart rate during the orthostatic challenge, thus confirming the negative effect of the low stroke volume that causes the narrow pulse pressure impairing diastolic capillary perfusion and suggesting a negative role of tachycardia [57]. Diastolic cardiac dysfunction is expected to strongly worsen the disturbance by impairing ventricular filling and stroke volume in the presence of a low filling pressure. In a recent study on patients suffering from COVID-19 infection, impaired left ventricular diastolic function and an impaired right ventricular function were echocardiographically detected [58]. Even physiological ventricular hypertrophy, as it occurs in sportsmen, might worsen ventricular filling under the condition of a low filling pressure. Tachycardia, as in POTS, can easily aggravate the disturbance due to a shortened filling time and reduced cardiac preload, which occur in a situation of low filling pressure. Frequent but short pressure peaks in tachycardia will not sufficiently accelerate and move forward a stagnating capillary blood column and will favor capillary stasis during diastole. A pharmacological confirmation of the negative role of tachycardia is the beneficial effect of the bradycardic drug Ivabradine in PCS patients [59,60,61,62]. None of the single hemodynamic disturbances outlined need to be strong enough to explain the severity of the disease, but all these hemodynamic disturbances acting in concert can cause transient capillary ischemia, the maldistribution of capillary blood flow, and can induce a peculiar type of flow disturbance, namely capillary ischemia/reperfusion. This also explains the lack of a characteristic diagnostic hemodynamic parameter. Capillary ischemia/reperfusion has a particular potential for triggering the development of mitochondrial dysfunction in skeletal muscle, as will be outlined in the next section.

## 5. Mitochondrial Dysfunction

**Figure 2:**
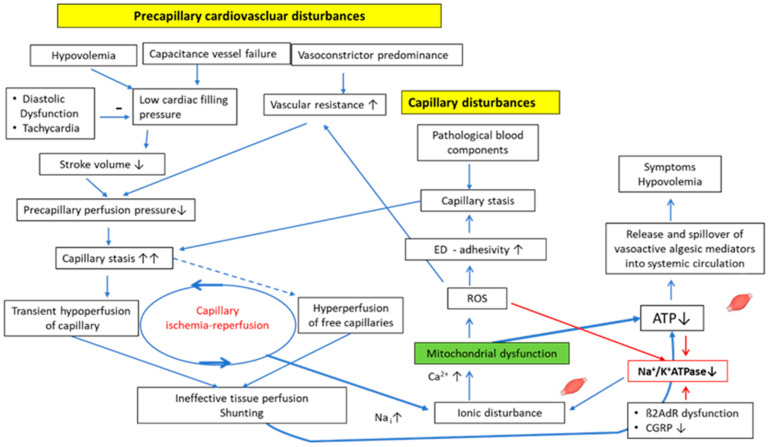
Proposed mechanisms of induction of the mitochondrial dysfunction as a result of the combined effect of perfusion disturbances, which are already shown in Figure 1, and an insufficient rise in the Na^+^/K^+^-ATPase activity in skeletal muscle. Mitochondrial dysfunction impairs perfusion via ROS and favors itself by lowering the Na^+^/K^+^-ATPase activity via ROS and low ATP. The poor energetic situation leads to the excessive production and spillover of vasoactive algesic mediators that can reach every organ to cause symptoms and induce hypovolemia. ED: endothelial dysfunction. Red arrows: strong inhibitory effect. Red box: The Na/K ATPase has a key role for the cellular homeostasis. (−) means inhibition. 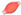 The symbol stands for skeletal muscle.

Energy depletion in skeletal muscle in ME/CFS is the consequence of the combined effect of perfusion disturbances and mitochondrial dysfunction, according to our hypothesis (Figure 2). This can explain low oxygen consumption on CPET, which can be considered a biomarker in patients with ME/CFS [63]. Finally, both disturbances trigger each other, as will be outlined in this section. First, we explain how the perfusion disturbances could trigger mitochondrial dysfunction in skeletal muscle and why the particular type of perfusion disturbance, namely capillary ischemia/reperfusion, plays an important role.

We have already used the experimental ischemia/reperfusion injury paradigm to explain mitochondrial dysfunction in a previous publication [64]. The recognition that capillary ischemia/reperfusion could occur as the consequence of the recently recognized capillary flow disturbances now allows us to more precisely work out the pathomechanisms involved and to recognize possible differences. In both conditions, it is finally sodium overload that indirectly causes damage. At a certain level of sodium, called the reverse mode threshold, the sodium–calcium exchanger (NCX) changes its transport direction importing calcium instead of exporting it. This leads to calcium overload and cellular and mitochondrial damage. We do not claim that the mechanisms of sodium loading are the same in all aspects in both conditions of ischemia/reperfusion, although we have identified capillary ischemia/reperfusion as the type of perfusion disturbance with the highest potential to cause mitochondrial dysfunction. We assume that a number of different disturbances—not only vascular disturbances—jointly raise intramuscular sodium in PCS and ME/CFS to finally cause calcium overload and damage. Capillary ischemia/reperfusion in ME/CFS is certainly less severe than the experimental ischemia/reperfusion situation in which a large vessel is totally occluded for a short time. In the classical experimental ischemia/reperfusion injury paradigm, a sudden and massively activated sodium–proton exchanger (NHE1), during earliest reperfusion, leads to extremely fast sodium loading and overload. There is overt tissue damage in the classical ischemia paradigm and mitochondrial dysfunction, while in ME/CFS there is no such tissue damage but evidence of mitochondrial disturbance. The experimental ischemia/reperfusion paradigm is enforced in general anesthesia, which causes tissue damage. Awake ME/CFS patients, however, would stop muscle work at a degree of effort that would cause clear tissue damage, so that mainly mitochondrial dysfunction occurs. In the experimental ischemia/reperfusion injury paradigm, the short occlusion of a vessel would not already cause damage by the short ischemia itself. It is sudden reperfusion that causes damage by sodium-induced calcium overload [65,66]. During total vascular occlusion, intravascular protons accumulate to almost totally inhibit NHE1 so that it cannot export protons any more against the high extracellular proton concentration that has accumulated in the occluded vessel during ischemia. With reperfusion, vascular proton concentration suddenly becomes normal. The NHE1, which is driven only by ion gradients, then becomes unleashed and extremely active for a short time only. It is then strongly driven by the high intracellular proton concentration generated by anaerobic metabolism during ischemia and accumulated in the cell due to the inhibition of the NHE1 by the high proton concentration in the totally occluded vessel. In early reperfusion, the transiently very high NHE1 activity causes sodium loading, in seconds rather than minutes. The sodium–potassium ATPase (Na^+^/K^+^ ATPase), which exports intracellular sodium for the import of potassium [67], cannot cope with such a sudden high sodium load—its activity is impaired by low ATP due to ischemia—so that sodium rises to cross the reverse mode threshold of the NCX to cause calcium overload for a brief moment. This is sufficient to cause damage.

A rise in intracellular sodium has indeed been demonstrated in skeletal muscles of patients with ME/CFS in an MRI study with 23-Na^+^ [68]. Depending on the muscle type, sodium was already elevated at rest before exercise or was found elevated only after exercise. Undoubtedly, continuous malperfusion already contributes to sodium loading via anaerobic metabolism to which mitochondrial dysfunction contributes as soon as it has developed. Anaerobic metabolism raises cellular proton production, which is extruded by NHE1. This causes continuous sodium influx and raises intracellular sodium. The reperfusion-induced sodium loading strongly adds to the metabolically and hypoperfusion-induced intracellular rise in sodium to cause or predispose to calcium overload. Presumably, enhanced sodium loading is not enough to cause a rise in sodium to an extent that it causes calcium overload by reversing the transport mode of the NCX. Another disturbance is needed.

In ME/CFS, intramuscular sodium loading is strongly aggravated by a disturbed function of Na^+^/K^+^-ATPase, diminishing cellular sodium export. The latter physiologically exports three sodium ions for the import of two potassium ions [67].

Thus, due to the disturbed function of Na^+^/K^+^-ATPase, the intramuscular sodium can already rise as soon as cellular sodium influx occurs as is the case at the beginning of exercise (via sodium-dependent transport processes such as via NHE1 and by the opening of sodium channels during the process of excitation).

During muscle work, Na^+^/K^+^ ATPase requires 10–20-fold stimulation as there is a strong sodium import by excitatory sodium channels and ion transport mechanisms [67,69]. The only hormonal stimuli of the Na^+^/K^+^-ATPase in the exercising muscle, apart from the rising sodium concentration itself, are ß2-adrenergic receptors (ß2AdR) and calcitonin-gene related peptide (CGRP) [67,69,70]. Deficiency of the only two mediators—ß2AdR and CGRP—that activate Na^+^/K^+^-ATPase during exercise can be considered a major cause of exercise intolerance, in conjunction with perfusion disturbances and mitochondrial dysfunction whereby all these three disturbances are interrelated in vicious cycles. ß2AdR dysfunction is assumed to be present in ME/CFS due to autoantibodies and/or desensitization to chronic stress in ME/CFS (high sympathetic tone) as the ß2AdR is the most sensitive adrenergic receptor to desensitization [71].

Small fiber neuropathy (SFN) occurs in part of the ME/CFS and PCS patients [18,72,73,74]. The sensory nerves that contain and release neuropeptides such as CGRP are primarily unmyelinated sensory C-fibers and myelinated A*δ*-fibers [75]. The importance of CGRP, and its release from sensory nerves, for the activation of the Na^+^/K^+^-ATPase and for diminishing muscle fatigue has been experimentally demonstrated under conditions of stimulation and inhibition [76,77]. After the degeneration of these fibers, by which CGRP is produced, there should be a deficiency in CGRP that adds to the stimulatory deficit of the Na^+^/K^+^-ATPase during exercise, which is already caused by ß2AdR dysfunction to aggravate and fix the disturbance. These neuropeptides released from the sensory nerves like Substance P and CGRP are also vasodilatory, so that their deficit as a result of SFN may contribute to muscular malperfusion. Deficiency in CGRP is certainly no necessity because only part of the patients develop SFN. However, once SFN has developed, it may contribute to fixing the disturbance. Na^+^/K^+^ ATPase activity is not only insufficiently activated during exercise but is even inhibited. At mitochondrial calcium levels higher than required for the stimulation of ATP production, the mitochondrion produces reactive oxygen species (ROS) [78]. ROS are generated as a consequence of mitochondrial dysfunction, and reduced ATP levels may further weaken mitochondrial activity [67,69]. Increased ROS production with evidence for inhibition of the Na^+^/K^+^ ATPase activity was indeed found in patients with ME/CF [79]. Cortisol, aldosterone, and triiodothyronine (T3) stimulate the expression of the Na^+^/K^+^ ATPase [69]. In addition, cortisol and aldosterone are in the low to normal range in ME/CFS and PCS [80,81,82].

Finally, we explain how mitochondrial dysfunction affects perfusion to close another vicious cycle of mutual triggering. Mitochondrial dysfunction produces ROS to cause endothelial dysfunction and to promote endothelial cell inflammation, the activation of coagulation, and adhesion [83]. The latter enhances vasoconstrictor influences and favors microcirculatory flow disturbances to further impair perfusion. Finally, via low ATP levels and the generation of ROS that inhibit Na^+^/K^+^-ATPase [84], and via anaerobic metabolism which produces more protons to raise intracellular sodium due to the NHE1, mitochondrial dysfunction favors itself [14,85,86,87]. The energetic disturbance is not severe enough to cause organ damage but limits a rise in physical and mental performance (exercise intolerance). Even worse, at a certain level of exercise, the individual post-exertional malaise threshold (PEM) threshold, intracellular sodium in skeletal muscle rises to reach the reverse mode threshold of the NCX. This causes calcium overload to trigger and renew mitochondrial damage. Thus, during exercise, the functional damage reproduces itself, keeping the patients captured in a vicious cycle from which they can hardly escape. Mitochondrial dysfunction can lead to reduced oxygen extraction, mimicking the picture of vascular shunting [18].

## 6. The Consequences of Critical Energetic Situation in Skeletal Muscle

The critical energetic situation in skeletal muscle leads to an excessive metabolically driven local generation of vasodilatory tissue mediators with algesic and inflammatory properties for a compensatory rise in skeletal muscle blood flow [14]. Mediators like bradykinin, prostaglandins, prostacyclin, and adenosine raising blood flow are physiologically meant to act locally only due to their very short half-lives. However, due to their excessive production following a poor metabolic situation in the large body’s muscle mass, spillover into the systemic circulation occurs. Any organ can be reached by them to produce a confusing myriad of symptoms including pain, spasms, and edema as a result of physiological actions. These mediators also cause renal hyperexcretion and induce microvascular leakage causing hypovolemia (see Section 2) (Figure 2) [14]. Their particular physiological renal actions prevent a compensatory rise in renin for repletion of the vascular system, thereby explaining the paradox that renin does not rise with hypovolemia [81]. This leads to a low stroke volume, as explained above, and causes orthostatic stress. The latter may be the greatest stressor that is involved in aggravating autonomic dysfunction and in the desensitization of ß2AdR to close the vicious circle. These vasodilator mediators released from skeletal muscle into the circulation can inappropriately dilate blood vessels in other organs (apart from the kidney) during exercise, at the expense of the working skeletal muscles (steal effect), to explain the observed left-to-right shunting and higher cardiac output during exercise in a group of patients [18].

## 7. Conclusions

The most severe complication of the Post-COVID-19 Syndrome (PCS) is the development of ME/CFS. Even in the absence of the full criteria defining ME/CFS, PCS and ME/CFS show a broad overlap in the symptomatology so that common mechanisms can be assumed to be operative. We hypothesize that precapillary cardiovascular disturbances that include hypovolemia and the failure of the capacitance vessels to adequately contract act in concert to reduce ventricular filling and thereby stroke volume. This, together with the predominance of vasoconstrictor mechanisms, which excessively constrict resistance vessels, reduces tissue perfusion and diminishes the capillary perfusion pressure to severely aggravate a preexisting capillary stasis. Capillary stasis is primarily caused by malformed blood components, microclots, and prothrombotic capillary wall changes as well as their concerted interactions. Vascular and capillary disturbances are favored by excessive reactive oxygen species (ROS) production and oxidative stress resulting from mitochondrial dysfunction. They reduce the availability of the vasodilator nitric oxide, affect the endothelial cellular barrier, and enhance endothelial adhesivity. Both precapillary and capillary disturbance have a highly synergistic detrimental effect on tissue perfusion. Together, they cause capillary ischemia and reperfusion, which favor the development of mitochondrial dysfunction in skeletal muscle and poor tissue perfusion. These disturbances are not severe enough to cause organ damage, but they prevent an adequate rise in physical and mental performance causing exercise intolerance. Even worse, when attempting to raise the level of effort and crossing the individual post-exertional malaise (PEM) threshold, the functional disturbances that cause these limitations in exercise capacity are triggered and renewed. Thus, the functional damage reproduces itself, keeping the patients captured in a vicious cycle from which they can hardly escape.

## Data Availability

Data sharing is not applicable to this article.
